# *Hippophae rhamnoides* L. Fruit Extract Relieves Chronic Idiopathic Constipation and Improves Bowel Function: A Monocentric, Randomized, Double-Blind, Placebo-Controlled, Clinical Trial

**DOI:** 10.3390/nu18050806

**Published:** 2026-02-28

**Authors:** Maria Vittoria Morone, Gaia Spadarella, Alessandro Di Minno, Marcello Cordara, Angela Cerqua, Lorenza Francesca De Lellis, Daniele Giuseppe Buccato, Alessandra Baldi, Roberto Piccinocchi, Hammad Ullah, Gaetano Piccinocchi, Xiang Xiao, Roberto Sacchi, Maria Daglia

**Affiliations:** 1 Department of Pharmacy, University of Napoli Federico II, 80131 Naples, Italyangycerqua@libero.it (A.C.); alessandra.baldi.alimenti@gmail.com (A.B.); 2 Department of Advanced Biomedical Sciences, University of Naples Federico II, 80131 Naples, Italy; 3 School of Medicine, University of Milano-Bicocca, 20126 Milan, Italy; 4 Anaesthesia and Resuscitation A. U. O. Luigi Vanvitelli, 80138 Naples, Italy; 5 School of Pharmacy, University of Management and Technology, 54000 Lahore, Pakistan; 6 Comegen S.C.S., Società Cooperativa Sociale, 80125 Naples, Italy; gpiccino@tin.it; 7 School of Food and Biological Engineering, Jiangsu University, 212013 Zhenjiang, China; 8 Applied Statistic Unit, Department of Earth and Environmental Sciences, University of Pavia, 27100 Pavia, Italy; 9 International Research Center for Food Nutrition and Safety, Jiangsu University, 212013 Zhenjiang, China

**Keywords:** *Hippophae rhamnoides* L., metabolic profiling, functional constipation, randomized clinical trial, constipation symptoms

## Abstract

**Background/Objectives**: Chronic idiopathic constipation (CIC) is a common gastrointestinal disorder with a global prevalence of about 14%, common in women and elderly population. It often lacks effective treatment. This randomized clinical trial was aimed to evaluate the efficacy and tolerability of *Hippophae rhamnoides* L. (sea buckthorn) fruit extract in adults with CIC. **Methods**: A UHPLC-HRMS/MS analysis was performed on the hydroethanolic *H. rhamnoides* fruit extract to evaluate its composition. Ninety participants were randomly assigned to receive either 500 mg of *H. rhamnoides* extract or placebo delivered through a capsule daily for 28 days. The primary outcome was the change in weekly spontaneous complete bowel movements (SCBMs), while secondary outcomes included stool consistency (Bristol Stool Form Scale—BSFS), gastrointestinal symptoms, and quality of life (SF-12). **Results**: Metabolic profile of the extract tentatively identified 75 bioactive compounds, predominantly flavonoids, triterpenoids and phospholipids. *H. rhamnoides* fruit extract significantly improved SCBM frequency (from 1.5 to 2.6 per week; *p* < 0.001) and normalized stool consistency (mean BSFS score from 1.4 to 3.5; *p* < 0.001), compared to no change in the placebo group. Significant reductions in bloating, abdominal pain, and heaviness were observed, while flatulence showed no between-group significant difference. No adverse events or use of rescue treatments were reported. Quality-of-life scores remained largely unchanged, with a non-significant trend towards improved mental health in the treated group. **Conclusions**: These findings suggest that *H. rhamnoides* fruit extract is a safe and effective option for managing CIC, offering an alternative to other plant extracts with laxative effects.

## 1. Introduction

As reported in the Rome Foundation Global Study published in 2021, chronic idiopathic constipation (CIC) is a common gastrointestinal disorder that affects approximately 11.7% of the global population, with a higher prevalence in females (15.2%) compared to males (8.3%). The incidence of CIC also varies by age: it is 13.2% in individuals aged 18 to 39 years, 11.0% in those aged 40 to 64 years, and 9.4% in individuals older than 64 years. This condition significantly impacts patients’ quality of life [1,2]. It is characterized by a spectrum of symptoms, including hard or lumpy stools, abdominal discomfort, infrequent or difficult bowel movements, and a sensation of incomplete evacuation. CIC represents a distinct subtype that typically arises in the absence of anatomical abnormalities or underlying systemic disease. It is primarily associated with poor dietary habits, psychological stress, and disturbances in the composition and function of the intestinal microbiota (dysbiosis) [2]. The main therapeutic goal in CIC management is to restore normal intestinal motility and bowel function and alleviate symptoms. Current clinical practice focuses on several approaches such as dietary modifications, and pharmacological and non-pharmacological treatments. Among these, laxative drugs are considered the first-line treatments. However, symptom relief is often temporary, and discontinuation of medication may result in symptom recurrence or worsening [3,4]. Despite the wide availability of over-the-counter laxatives, constipation remains a challenging condition to manage. In the United States, the direct annual healthcare cost per patient has been estimated at USD 7522. In 2014, Dik et al. reported that, due to a lack of studies, the impact of CIC on healthcare budgets in Western Europe is unknown [5]. More recently, in the UK, the cost of treating constipation by the English National Health Service was £162 million in 2017–2018. This highlights the significant economic burden and the need for more effective and sustainable treatment strategies [5,6,7].

In this context, increasing attention has been directed toward the use of vegetable extracts due to their broad beneficial effects in alleviating CIC symptoms [8]. Nevertheless, the traditional use of certain botanical species, such as *Aloe vera* (L.) Burm.f., *Senna alexandrina* Mill., and *Frangula purshiana* (DC.) A.Gray ex J.G.Cooper, has raised safety concerns in Europe. The European Food Safety Authority (EFSA) is currently evaluating the safety of these plants because they contain hydroxyanthracenes which exert carcinogenic activity [9]. *Hippophae rhamnoides* L., commonly known as sea buckthorn, has been used in traditional medicine for centuries to address a wide range of health conditions, including respiratory ailments, gastrointestinal disorders, circulatory problems, and pain relief [10]. Its leaves, rich in tannins, have been employed in the management of diarrhea, while fruit extracts have demonstrated therapeutic potential against hepatic, respiratory, metabolic, and gastrointestinal illnesses [11,12,13]. These effects are attributed to its content of nutrients and bioactive compounds, including fat-soluble vitamins, vitamin C, organic acids, polyphenols, terpenes, carbohydrates, lipids, amino acids, and dietary fibers [14]. In 2019, a study investigated the properties of a sea buckthorn fruit extract in constipation using ex vivo and in vivo model systems. The findings revealed that *H. rhamnoides* fruit extract enhanced fecal output and exerted both laxative and prokinetic effects, primarily through the activation of muscarinic receptors [15]. More recently, we investigated the effect of *H. rhamnoides* fruit dry extract on Aquaporin-3 (AQP-3) expression in the intestinal epithelial cell line (HT-29), which is hypothesized to play a key role in water transport in the colon, showing that the extract significantly upregulated AQP-3 expression in the absence of cytotoxicity. In addition, after in vitro simulated digestion and fermentation with gut microbiota isolated from healthy and constipated subjects, the *H. rhamnoides* fruit extract induced an increase in gut microbiota functionality shown by the increase in the production of short-chain fatty acids (SCFA) [16].

Building on the promising results from previous preclinical investigations, this clinical study was designed to evaluate the efficacy and tolerability of sea buckthorn fruit extract for improving gastrointestinal health in subjects with CIC, characterized by symptoms including bloating, abdominal distension, heaviness, abdominal pain, and flatulence.

## 2. Materials and Methods

### 2.1. H. rhamnoides Fruit Extract and Placebo

The commercially available *H. rhamnoides* fruit extract was obtained by EPO S.r.l. (Via Stadera 19, 20141 Milan, Italy) starting from harvested and dried sea buckthorn fruit, submitted to hydroethanolic maceration, followed by percolation, under vacuum concentration, atomization through spray-drying, ground and sieved through multiple sieves of decreasing mesh size to obtain a fine powder. The dried hydroethanolic *H. rhamnoides* fruit extract was standardized by isorhamnetin. In the clinical trial, to deliver this extract at the daily dose of 500 mg, each capsule contained 500 mg of *H. rhamnoides* fruit dry extract (titrated to 0.1% isorhamnetin), hydroxypropylmethylcellulose (E464) as coating agent, magnesium salts of fatty acids (E470b) as anti-caking agent, and iron oxides and hydroxides (E172) as colouring agent. The placebo consisted of the inert ingredients (E464, E172, and E470b) and maltodextrin, and was identical in appearance, color, odor, taste, weight, and packaging to the food supplement, thereby ensuring blinding. Both the treatment and the placebo were manufactured by FMC S.r.l. (Via Asi Consortile, 03013 Ferentino, FR, Italy) in accordance with the European specifications for contaminants and microbiological limits. Moreover, the clinical trial treatment was registered with the Italian Ministry of Health (notification number: 2025/194153) in accordance with the Italian Legislative Decree 169/2004 and Ministry of Health guidelines [17], which require that clinical trials on foods and food ingredients focus on food products that fully comply with existing food regulations. The study products were supplied free of charge by EPO S.r.l. (Via Stadera 19, 20141 Milan, Italy), sponsor of the clinical trial.

### 2.2. RP-UHPLC-ESI-Orbitrap-MS/MS Analysis

#### 2.2.1. Sample Preparation

The powdered *H. rhamnoides* extract sample (100 mg) was solubilized with methanol solution (1 mL) for 30 min at 25 °C. Then, the mixture was centrifugated for 15 min at 6000 rpm at 4 °C and the obtained supernatants were transfer into LC vials and injected into RP-UHPLC-ESI-Orbitrap-MS/MS system.

#### 2.2.2. RP-UHPLC-ESI-Orbitrap-MS/MS Conditions

UHPLC-HRMS/MS analysis was performed on a Thermo Scientific™ Vanquish™ UHPLC system (Milan, Italy), equipped with a VF-P10-A binary solvent delivery system, a VC-D11-A photodiode array detector, a VH-C10-A column compartment and VF-A10-A autosampler. The UHPLC system was coupled online to a Orbitrap Exploris 120 mass spectrometer (Thermo Fisher Scientific, Bremen, Germany) equipped with a heated electrospray ionization probe (HESI II) operating in negative and positive mode.

The chromatographic separation was performed on a Kinetex^®^ 2.6 µm EVO C18 100 Å, LC Column 150 × 2.1 mm (Phenomenex, Bologna, Italy). The column temperature and the flow rate were set at 40 °C and 0.4 mL/min, respectively. The mobile phases were H_2_O (A) and ACN (B) both acidified with 0.1% HCOOH (*v*/*v*) with the following gradient: 0.01–17.00 min, 2–95% B; 17.01–19.00 min, isocratic to 95% B; 19.01–20.00 min, 95–2% B; then five minutes for column re-equilibration.

The MS was calibrated by Thermo Pierce™ FlexMix™ Calibration Solutions (Milan, Italy) in both polarities. Full MS (100–1500 *m*/*z*) and data-dependent MS/MS were performed at a resolution of 60,000 and 15,000 FWHM, respectively; Normalized Collision Energy (NCE) value of 30 was used. Source parameters: Sheath gas pressure, 40 arbitrary units; auxiliary gas flow, 15 arbitrary units; spray voltage, +3.0 kV, −2.0 kV; capillary temperature, 320 °C; auxiliary gas heater temperature, 300 °C.

The tentative identification of the investigated analytes was carried out by comparing their retention times and MS/MS data with those present in the literature. For data analysis, Compound Discoverer^TM^ 3.1 software (Thermo Scientific, SanJose, CA, USA) was used for raw data processing (baseline correction, noise filtering, spectral alignment, and peak detection) and for putative identification of metabolites based on molecular formula (matched), exact mass (mass tolerance < 5 ppm) and MS2 fragmentation pattern [Fragment Ion Search (FISh)], with a global database search (mzCloud, MassList and ChemSpider).

### 2.3. Clinical Trial Design

A monocentric, randomized, double-blind, placebo-controlled, two-arm, parallel-group clinical trial was conducted by the principal investigator, a general practitioner with a clinical trial qualification in accordance with current legislation, at COMEGEN Soc. Coop. Sociale (Naples, Italy) to evaluate the efficacy of *H. rhamnoides* fruit extract in adults with chronic idiopathic constipation recruited in an outpatient setting by the principal investigator. The intervention period lasted four weeks, a duration considered adequate by the European Food Safety Authority (EFSA) [18] for assessing the efficacy of foods or food ingredients on intestinal function. Participants were randomly assigned to one of two groups: the *H. rhamnoides* group that received 1 capsule at the daily dose of 500 mg of *H. rhamnoides* fruit extract, corresponding to 0.5 mg isorhamnetin, for 28 days and the placebo group that received one placebo capsule daily. Each participant underwent evaluation of primary and secondary outcomes both before (T0) and after (T1) the 28-day treatment period.

The study was conducted in accordance with the ethical principles of the Declaration of Helsinki (1964, and subsequent amendments up to Fortaleza, 2013) and Good Clinical Practice guidelines (CPMP/ICH/135/95). All participants provided written informed consent prior to any study-related procedure. The study protocol, informed consent form, and related documentation were reviewed and approved by the Ethics Committee Campania 1 (Protocol No. 93, 3 April 2025). The Clinical trial registration was done on ClinicalTrials.gov (ID NCT07082673—date of registration 23 July 2025).

The study consisted of two main visits: baseline (T0) and follow-up (T1, after 28 days of treatment). At T0, the general practitioner’s patients, preliminarily identified as potentially eligible subjects, were screened based on inclusion and exclusion criteria. In more detail, screening procedures also included an HIV fourth-generation rapid saliva test, detecting both anti-HIV antibodies and the p24 antigen, and a urine pregnancy test (β-hCG) for women of childbearing potential (subjects with indeterminate or positive test results were excluded). During the same visit, the subject who met the inclusion and exclusion criteria signed the informed consent form. Then, the data on the assessment of bowel function (i.e., number of spontaneous complete bowel movements (SCBM), stool consistency (Bristol Stool Form Scale, BSFS), gastrointestinal symptoms (bloating, distension, heaviness, pain, flatulence), use of rescue treatments, and quality of life (SF-12 questionnaire), during the previous four weeks), and the measurements of body mass index (BMI) and waist circumference (WC) were collected and reported in the case report form (CRF). Then, the recruited subjects were randomized (allocation ratio 1:1) into one of the two study groups using computer-generated randomization. To ensure an adequate balance between sexes across treatment arms, randomization was stratified by sex. The allocation sequence was generated by an independent researcher not involved in enrolment or assessment. The assigned study products (*H. rhamnoides* fruit extract or placebo) and a bowel function diary were provided.

At T1, participants returned for the follow-up visit after 28 days of treatment. The bowel function diary was collected, and primary and secondary outcomes were reassessed based on patient-reported data for the preceding four weeks. Compliance was verified through capsule counts, and participants were interviewed regarding product tolerability and the occurrence of any adverse events. To minimize possible biases in data interpretation, participants were provided with the following instructions: (i) defecation posture (proper defecation posture was demonstrated at baseline (T0) using a pictogram), (ii) fluid intake (participants were instructed to consume approximately 2 L of fluids per day and record intake in the bowel function diary), (iii) concomitant treatments (all pharmacological treatments taken during the study were recorded at each visit), (iv) dietary habits (participants were asked not to modify their habitual diet throughout the study period).

### 2.4. Inclusion and Exclusion Criteria

Participants of both sexes were eligible for inclusion if they met the following criteria: age 18–70 years; ability to understand and sign informed consent; negative HIV and pregnancy tests (where applicable); presence of chronic idiopathic constipation symptoms for ≥3 months, with onset ≥ 6 months prior to screening; absence or non-predominance of abdominal pain (less than one day per week); fewer than three spontaneous complete bowel movements per week, and at least one of the following (straining during >25% of defecations, lumpy or hard stools (BSFS type 1 or 2) in >25% of defecations, sensation of incomplete evacuation in >25% of defecations, sensation of anorectal blockage in >25% of defecations, and manual maneuvers required for evacuation in >25% of defecations); not currently using, and agreeing not to use, medications affecting bowel function; willingness and ability to comply with study procedures.

Participants were excluded if they met any of the following criteria: age < 18 or >70 years; pregnancy or breastfeeding; abdominal pain at least once per week (IBS-C diagnosis); presence of organic intestinal disease; history of gastrointestinal surgery; gastroesophageal reflux disease; neurodegenerative diseases (e.g., Parkinson’s, Alzheimer’s disease); HIV-positive or immunocompromised; any medical condition considered incompatible with participation; use of opioids or medications significantly affecting bowel function; recent antibiotic therapy (within the last 4–6 weeks); use of chronic medications for other diseases; alcohol, drug, caffeine, or theine abuse; cognitive impairment or non-self-sufficiency; difficulty attending study visits or unwillingness to cooperate; known allergy to any component of the investigated or placebo product.

### 2.5. Outcomes of Study

The primary outcome of this clinical study was to evaluate the efficacy of *H. rhamnoides* fruit extract at the daily dose of 500 mg, in improving the frequency of spontaneous complete bowel movements (SCBMs), which are defined by the European Medicines Agency (EMA) [19] as an appropriate primary outcome in the clinical evaluation of active substances for chronic idiopathic constipation. This outcome incorporates the concept of spontaneity, meaning no use of any salvage treatment (medications or any other laxatives, including dietary supplements, enemas, or suppositories) within 24 h prior to the bowel movement, as well as the completeness of the evacuation. Mean number of SCBMs per week in the previous month: (i) reported by the subject at the recruitment visit (T0) and recorded by the investigator in the Case Report Form (CRF), and (ii) calculated at the final visit (T1) based on entries in the subject’s bowel function diary. The use of a daily diary, as recommended by EMA guidelines, helps to avoid recall bias that may occur at clinic visits (T0 and T1).

Secondary outcomes of the study included stool consistency, severity of constipation symptoms, use of salvage treatment, and quality of life (QoL) during the past four weeks. Stool consistency was assessed at T0 and T1 using Bristol Stool Form Scale (BSFS), classifying stool consistency into seven types. Types 1 and 2 indicate hard or lumpy stools, while types 6 and 7 correspond to soft or watery stools. Individuals with constipation typically have type 1 or 2 stools. Severity of characteristic constipation symptoms include bloating and abdominal distension, feeling of heaviness, abdominal pain, and flatulence, reported by subjects during visits and throughout the study period via the bowel function diary. Salvage treatment was recorded in the bowel function diary. Symptoms were assessed using a 5-point Likert scale. For each item, participants rated the intensity or frequency of [20]. Subjects were instructed to record any use of salvage treatment (medications or any other laxatives, including dietary supplements, enemas, or suppositories) intended to improve bowel function. Use of such treatments did not automatically lead to exclusion from the study or interruption of experimental treatment; however, whether to include or exclude data from such subjects in the final analysis was at the principal investigator’s discretion. Participants were informed before treatment initiation that salvage treatment could be used if documented in the diary and if they experienced a reduction of at least one SCBM per week. Impact of constipation on perceived QoL during the past four weeks was assessed at T0 and T1 using Short Form Health Survey-12 (SF-12), a validated questionnaire developed through multi-year studies in patients with chronic conditions, widely used in clinical practice for self-assessment of quality of life in relation to general health disorders.

### 2.6. Safety

*H. rhamnoides* fruit extract treatment and placebo were composed exclusively of legally approved food-grade ingredients with a well-established safety record in the EU. The *H. rhamnoides* fruit extract is authorized under Italian food supplement regulations, and no safety alerts or adverse event reports are associated with its use. Although no adverse events were expected, participants were closely monitored throughout the study. Any suspected adverse reaction was to be reported to the national VigiErbe phytovigilance system [21] managed by the National Institute of Health (Italy).

In the unlikely event of a suspected unexpected serious adverse reaction (SUSAR), the incident would be documented in the CRF, promptly reported to the competent Ethics Committee, and recorded in the participant’s medical file. Concomitant medication uses and relevant clinical findings (including laboratory data) would be noted. Participants experiencing SUSARs would be withdrawn from the trial.

### 2.7. Data Collection

Data were collected using structured Case Report Forms (CRFs) divided into two main sections: (i) participant information, medical history, concomitant medication, and treatment allocation; (ii) primary and secondary outcome measurements. Adverse events were recorded using a dedicated form based on the template provided by the Italian National Institute of Health for reporting suspected adverse reactions to vegetable extracts.

### 2.8. Statistical Analysis

The power analysis was based on the interaction term between time and treatment, which assesses whether the change in SCBM frequency over time differs between the treatment and control groups. A repeated-measures ANOVA framework was adopted with one between-subject factor (Treatment: *H. rhamnoides* group and placebo group) and one within-subject factor (Time: T0, T1). A balanced design with equal observations per cell was assumed. As no prior data were available on the effect of *H. rhamnoides* fruit extract on SCBM frequency, conventional Cohen’s effect size estimates were adopted for small (f = 0.10), small-to-medium (f = 0.20), and medium (f = 0.25) effects. The within-subject correlation was set at 0.25. For a 95% power (1 − β = 0.95) and α = 0.05, with a small-to-medium effect size (f = 0.15), a minimum total sample size of 82 participants was required to detect a significant time × treatment interaction. To account for an estimated dropout rate of 10%, the final number of enrolled subjects was increased to 90 (*n* = 45 per group).

Descriptive statistics (mean, standard deviation, minimum–maximum range) were calculated for all variables within each treatment group. The normality of the variables was preliminarily assessed through graphical inspection (e.g., Q–Q plots and histograms). The primary and secondary outcome variables were analyzed using linear mixed-effects models (LMMs) with a random intercept to account for intra-subject variability. Each variable was entered as dependent variable in a separate model. Fixed effects included time (T0, T1), treatment (*H. rhamnoides* group and placebo group), and their interaction (time × treatment), with age and sex included as covariates. Subject identity was modelled as a random factor to control individual differences in treatment response. Analyses were conducted using the lme4 package [22] in R ver. 4.0.1 [23]. Degrees of freedom were estimated using the Satterthwaite approximation. Results are presented as means ± standard deviations unless otherwise indicated.

## 3. Results

### 3.1. Chemical Profile of H. rhamnoides Extract

**Figure 1:**
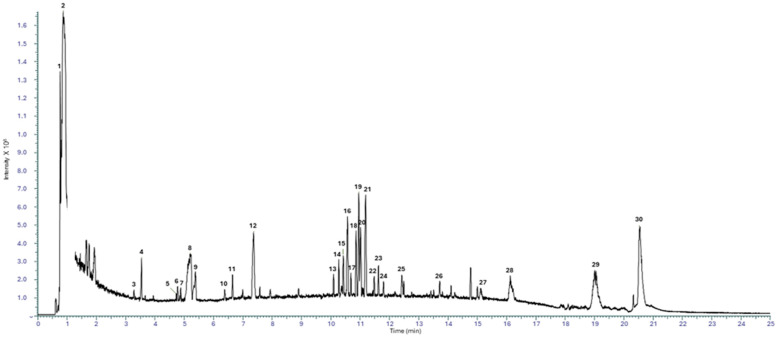
Representative TIC chromatogram in negative ionization mode.

**Figure 2:**
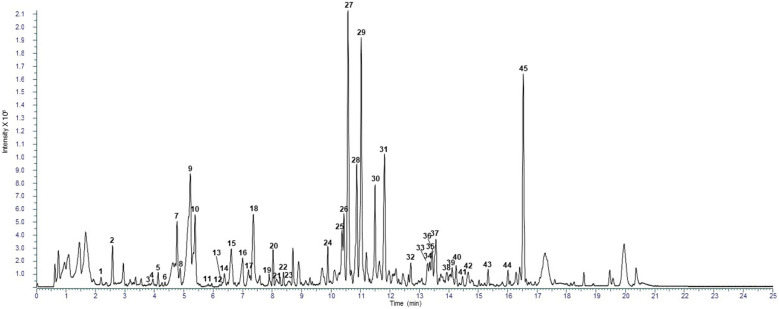
Representative TIC chromatogram in positive ionization mode.

Chemical characterization of the commercial hydroethanolic *H. rhamnoides* fruit extract was obtained using RP-UHPLC coupled with a Q Exactive hybrid quadrupole-Orbitrap mass spectrometer. Through a comparison with the in silico MS/MS spectra, accurate mass, and molecular formula, 75 compounds (45 for positive HESI II mode, and 30 for negative HESII mode) were tentatively annotated in *H. rhamnoides* fruit extract, respectively, with confidence MSI lvl.2 [24]. The tentative identification was reported in Table 1 and representative total ion current (TIC) negative and positive chromatograms were reported in Figure 1 and Figure 2, respectively.

### 3.2. Randomized Clinical Trial

**Figure 3:**
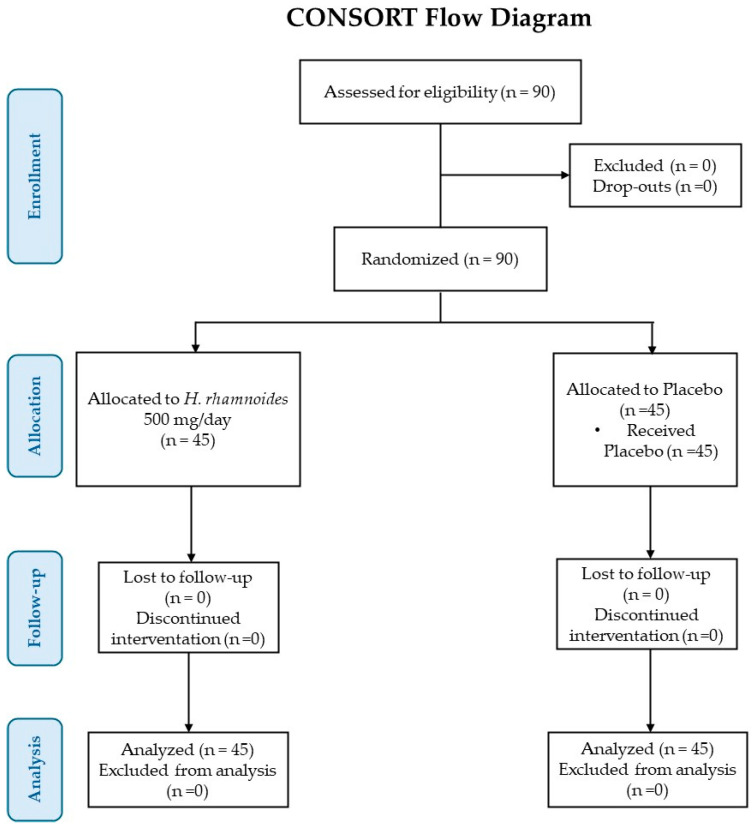
CONSORT flow diagram.

Figure 3 displays a study flowchart, produced following CONSORT PRO reporting guidelines [25]. Table 2 presents the baseline (T0) demographic and clinical data about the enrolled participants. The sample includes 45 subjects for each experimental group (25 women and 20 men for the placebo group, and 20 women and 25 men for the group treated with *H. rhamnoides* fruit extract). The mean age (±SD) of subjects was 44.3 ± 14 years for the placebo group and 43.8 ± 15 for the *H. rhamnoides* group.

Table 3 reports the descriptive statistics (mean, standard deviation, and range) for the 11 response variables measured from time T0 to time T1 in the two experimental groups.

**Figure 4:**
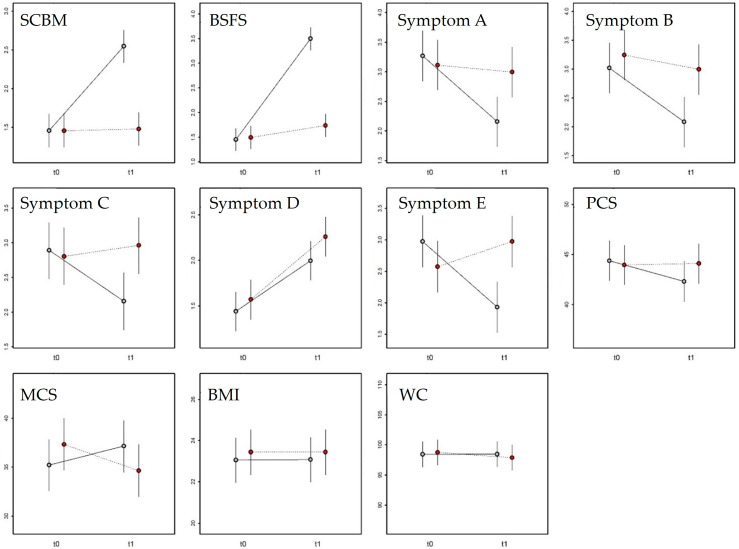
Comparison between the *H. rhamnoides* (grey) and placebo (red) groups for the 11 variables measured at time points T0 and T1, as predicted by the LMMs (means and 95% confidence intervals). SCBM: Number of bowel movements per week; BSFS: Bristol Stool Form Scale score; Symptom A: Bloating sensation; Symptom B: Abdominal distension; Symptom C: Heaviness; Symptom D: Flatulence; Symptom E: Abdominal pain; PCS: Quality-of-life score—physical component; MCS: Quality-of-life score—mental component; BMI: Body Mass Index; WC: Waist circumference.

The LMM for the SCBM score (Table 4) identified significant effects for treatment (*p* < 0.001) and measurement (*p* < 0.001), as well as a significant interaction between the two (*p* < 0.001). No significant effects were found for age or sex (Table 4). The results indicate that SCBM scores changed over time differently between the two experimental groups (Figure 4). Specifically, SCBM scores at T0 did not differ between groups (β = 0.0026 ± 0.14, t_174_ = 0.019, *p* = 0.98). After H. rhamnoides treatment, the SCBM score increased significantly at T1 (β = 1.09 ± 0.14, t_174_ = 7.759, *p* < 0.001), while it remained unchanged in the placebo group (β = 0.022 ± 0.14, t_174_ = 0.158, *p* = 0.87). At T1, the SCBM score was significantly higher in the H. rhamnoides group compared with placebo (β = 1.07 ± 0.14, t_174_ = 7.620, *p* < 0.001).

The LMM for the Bristol Stool Form Scale (Table 4) identified significant effects of treatment (*p* < 0.001) and measurement (*p* < 0.001), and a significant interaction (*p* < 0.001). A significant effect of age (*p* = 0.014) but not of sex was also observed (Table 4). The results indicate that the Bristol score changed over time differently between the two experimental groups (Figure 4). Specifically, at T0 the scores did not differ between groups (β = 0.04 ± 0.15, t_174_ = 0.265, *p* = 0.79). After treatment, the Bristol score increased significantly in the *H. rhamnoides* group at T1 (β = 2.04 ± 0.15, t_174_ = 13.398, *p* < 0.001) but remained unchanged in the placebo group (β = 0.24 ± 0.15, t_174_ = 1.602, *p* = 0.11). At T1, the Bristol score was significantly higher in the *H. rhamnoides* group than in the placebo group (β = 1.76 ± 0.15, t_174_ = 11.531, *p* < 0.001). Regardless of treatment, the score decreased significantly with increasing age (β = –0.13 ± 0.05, t_174_ = 2.478, *p* = 0.014).

The LMM for Symptom A, corresponding to bloating sensation, (Table 4) revealed significant effects of measurement (*p* < 0.001) and the interaction between measurement and treatment (*p* < 0.001), but not for treatment alone. No significant effects were found for age or sex (Table 4). The results indicate that the score for Symptom A varied over time differently between the two groups (Figure 4). At T0, there were no differences between groups (β = 0.15 ± 0.28, t_174_ = 0.574, *p* = 0.57). After treatment, the score for Symptom A decreased significantly in the *H. rhamnoides* group (β = 1.11 ± 0.28, t_174_ = 3.997, *p* < 0.001) but remained unchanged in the placebo group (β = 0.11 ± 0.28, t_174_ = 0.400, *p* = 0.69). At T1, the symptom score was significantly lower in the *H. rhamnoides* group than in the placebo group (β = 0.84 ± 0.28, t_174_ = 3.023, *p* = 0.0029).

The LMM for Symptom B, corresponding to abdominal distension, (Table 4) showed significant effects of measurement (*p* = 0.0041) and treatment (*p* = 0.0059), while the measurement × treatment interaction was near the significance threshold (*p* = 0.091). No effects were found for age or sex (Table 4). The results indicate that Symptom B scores varied between measurements and tended to diverge between groups (Figure 4). At T0, no differences were found (β = 0.22 ± 0.29, t_174_ = 0.766, *p* = 0.44). After treatment, the Symptom B score decreased significantly in the *H. rhamnoides* group (β = 0.93 ± 0.29, t_174_ = 3.260, *p* = 0.0013) but remained unchanged in the placebo group (β = 0.24 ± 0.29, t_174_ = 0.854, *p* = 0.39). At T1, the score was significantly lower in the *H. rhamnoides* group (β = 0.91 ± 0.29, t_174_ = 3.172, *p* = 0.0018).

The LMM for Symptom C, corresponding to heaviness, (Table 4) identified a significant interaction between measurement and treatment (*p* = 0.021), with no significant effects for age or sex. The results indicate that Symptom C scores changed differently over time between groups (Figure 4). At T0, scores did not differ between groups (β = 0.08 ± 0.27, t_174_ = 0.312, *p* = 0.75). After treatment, the Symptom C score decreased significantly in the *H. rhamnoides* group (β = 0.73 ± 0.27, t_174_ = 2.717, *p* = 0.0073), but not in the placebo group (β = 0.15 ± 0.27, t_174_ = 0.576, *p* = 0.56). At T1, the score was significantly lower in the *H. rhamnoides* group (β = 0.80 ± 0.27, t_174_ = 2.977, *p* = 0.0033).

The LMM for Symptom D, corresponding flatulence, (Table 4) identified a significant main effect of measurement (*p* < 0.001) but not of treatment or their interaction. No significant effects were observed for age or sex (Table 4). The results indicate that Symptom D scores changed similarly over time in both groups (Figure 4). At T0, scores did not differ (β = 0.13 ± 0.14, t_166_ = 0.926, *p* = 0.36). Following treatment, the Symptom D score increased significantly in both the *H. rhamnoides* group (β = 0.55 ± 0.14, t_88_ = 4.413, *p* < 0.001) and the placebo group (β = 0.69 ± 0.14, t_88_ = 5.423, *p* < 0.001). Accordingly, at T1, there was no significant difference between groups (β = 0.26 ± 0.14, t_166_ = 1.689, *p* = 0.063).

The LMM for Symptom E, corresponding to abdominal pain, (Table 4) identified a significant measurement × treatment interaction (*p* < 0.001), with no effects of age or sex. The results indicate that Symptom E scores changed differently between groups over time (Figure 4). At T0, no differences were observed between groups (β = 0.40 ± 0.27, t_174_ = 1.490, *p* = 0.14). After treatment, the score decreased significantly in the *H. rhamnoides* group (β = 1.04 ± 0.27, t_174_ = 3.866, *p* < 0.001), while it remained unchanged in the placebo group (β = 0.40 ± 0.27, t_174_ = 1.481, *p* = 0.14). At T1, the score was significantly lower in the *H. rhamnoides* group than in the placebo group (β = 1.04 ± 0.27, t_174_ = 3.856, *p* < 0.001).

The LMM for the Physical Component Summary (PCS) score of the SF-12 questionnaire (Table 4) did not identify any significant effects. The variable therefore did not differ between measurements or in response to treatment (Figure 4). Similarly, the LMM for the Mental Component Summary (MCS) score of the SF-12 questionnaire showed no significant effects for any variable (Table 4). The MCS score, thus, did not differ between groups or across time points (Figure 4). The LMM for Body Mass Index (BMI) revealed no significant effects for measurement, treatment, or their interaction (Table 4). A significant effect of sex (*p* = 0.029) but not age was observed. BMI therefore did not differ between groups or across time. Regardless of time and treatment, BMI values were significantly higher in women (β = 1.46 ± 0.66, t_86_ = 2.208, *p* = 0.030). The LMM for waist circumference (WC; Table 3) did not reveal any significant effects. Consequently, WC did not differ between measurements or in response to the two experimental treatments (Figure 4).

In addition, no subject reported the use of any salvage treatment, including laxative drugs, dietary supplements, enemas, or suppositories. The *H. rhamnoides* treatment was well-tolerated by all participants throughout the 28-day intervention period, as evaluated by the Principal Investigator.

## 4. Discussion

In this study, a chemically characterized *H. rhamnoides* fruit extract was evaluated for its efficacy in improving intestinal function, constipation symptoms, quality of life, and tolerability in subjects with chronic idiopathic constipation at two times (T0, baseline; T1, 28 days post-treatment).

The comprehensive profiling of *H. rhamnoides* fruit extract using RP-UHPLC-ESI-Orbitrap-MS/MS analysis revealed that the extraction process kept the phytocomplex intact with the presence of many phytochemicals [13], predominantly flavonoids (flavonols, particularly isorhamnetin and its glycosides, as well as quercetin and kaempferol derivatives, and a flavone, luteolin) followed by glycerophosphocholines, glycerophosphoethanolamines, and glycerophospholipids (PC, lysoPE and lyso PI), triterpenoids and organic acids (e.g., malic acid and quinic acid). These bioactive compounds, especially flavonoids, are known to exert health-promoting properties including prebiotic activity, which promotes intestinal function by helping to rebalance the gut microbiota composition and functionality [26].

Regarding the clinical trial, EFSA and EMA guidelines [18,19] were followed in the planning of the experimental design of this monocentric, randomized, parallel-group, double-blind, placebo-controlled clinical trial. The *H. rhamnoides* fruit extract at the daily dose of 500 mg for 28 days significantly improved the primary outcome, increasing SCBMs from 1.5 to 2.6 per week, whereas the placebo group showed no change. *H. rhamnoides* fruit extract also improves secondary outcomes of the study, supporting its potential benefit in functional constipation. Stool consistency improved from Bristol Scale type 1–2 to a mean of 3.5 (closer to normal), and improvement in BSFS indicated an overall amelioration of some of the constipation-related symptoms such as bloating sensation, feeling of heaviness, and abdominal pain. A non-significant trend towards improvement in the MCS was observed in the *H. rhamnoides* group, while the PCS remained unchanged. Furthermore, no subject reported the use of any salvage treatment, including laxative drugs, dietary supplements, enemas, or suppositories. The tested *H. rhamnoides* fruit extract was well tolerated by all participants, with no adverse events reported or observed.

Recently, Dimidi et al. [27] published comprehensive evidence-based dietary guidelines for the management of chronic constipation. These guidelines found inconclusive evidence for probiotic/symbiotic supplements, additional fluids (2 L/day), senna supplements, vitamin C, and caffeine, highlighting the need for further robust clinical studies to establish their efficacy and safety and the extension of the research to new effective and safe active ingredients. Positive results were observed only for psyllium, prunes, and kiwifruits, which are rich in dietary fibers; however, these interventions primarily improved specific symptoms rather than stool consistency or frequency. In this context, the present study addresses an important gap by evaluating the effects of an *H. rhamnoides* fruit extract on the number of spontaneous complete bowel movements per week, stool consistency, constipation symptoms, and quality of life in adults with functional constipation.

*H. rhamnoides* is quite rich in bioactive compounds such as polyphenols, omega fatty acids, phytosterols, vitamins (C, E, and K_1_), carotenoids, and bioactive polysaccharides, all of which are quite effective against digestive diseases [28]. Our results are in agreement with those published by Shijie et al. [29], who evaluated the clinical efficacy of a dried *H. rhamnoides* emulsion in the management of functional constipation in children. A 28 day-supplementation of subjects with dried emulsion resulted in improved constipation symptoms, stool frequency, and symptoms recovery rate. In another study, Hanif et al. [15] reported prokinetic, laxative, and gut excitatory effects of *H. rhamnoides* fruit extract in rodents, mediated through particle activation of muscarinic receptors. The study evaluated the prokinetic and laxative effects using crude fruit extract in mice, measuring intestinal transit of charcoal and fecal output. Low doses stimulated intestinal transit and significantly increased fecal elimination, whereas higher doses showed inhibitory effects on the gastrointestinal tract. The excitatory effects of *H. rhamnoides* were similar to those of carbachol, a cholinergic agonist that stimulates intestinal motility, suggesting that *H. rhamnoides* acts on muscarinic receptors, similar to acetylcholine, a key gastrointestinal neurotransmitter [30]. Additionally, when mice were treated with atropine, a muscarinic antagonist, the prokinetic and laxative effects of sea buckthorn were significantly reduced, indicating that the stimulatory action is partially mediated by muscarinic receptors. Phytochemical analysis of the *H. rhamnoides* fruit extract revealed the presence of 11 isorhamnetin derivatives, which may justify an increase in cholinergic activity because they exerts an inhibitory action of acetylcholinesterase preventing the degradation of acetylcholine (ACh) in the synaptic space and thus, causing ACh concentration to increase and prolonging its action on receptors [31]. The combination of excitatory and inhibitory components in the extract may provide a balanced effect, reducing side effects such as cramping, which are common with other parasympathomimetic drugs used to relieve constipation [32].

Moreover, our previous in vitro study evaluated the expression of aquaporin-3 (AQP3) in human colorectal adenocarcinoma cells (HT-29) after treatment with non-cytotoxic concentrations of this standardized dry fruit extract of *H. rhamnoides* enriched in isorhamnetin. AQPs are transmembrane proteins composed of six transmembrane helices and two non-transmembrane helices that form tetramers to facilitate water transport across cell membranes [33]. In mammals, classical aquaporins (AQP1, 4, and 5) transport only water, whereas aquaglyceroporins (AQP3 and 9) transport water and glycerol. AQP3, mainly expressed on the apical and basolateral membranes of colonic epithelial cells, is a protein of approximately 32 kDa. Numerous studies highlight the importance of AQP3 in intestinal water and fluid transport [33], and altered expression is associated with disorders such as diarrhea and/or constipation [34]. In constipation, osmotic laxatives, which draw water into the lumen to soften stools, are often prescribed, unlike stimulant or irritant laxatives that increase intestinal peristalsis. Although both types ultimately promote evacuation, they differentially affect AQP3 expression: osmotic laxatives such as magnesium sulfate, lactulose, and mannitol increase AQP3 expression in rat and mouse intestines, whereas stimulant laxatives, like bisacodyl and sennoside A, decrease AQP3 expression [34]. Therefore, aquaporins may represent a therapeutic target in disorders such as diarrhea or constipation. The aforementioned in vitro study indicated that at a concentration of 100 µg/mL, the dry fruit extract induced overexpression of AQP3 in HT-29 cells compared to untreated controls, suggesting that it acts via a mechanism similar to osmotic laxatives in constipation. The study also confirmed the safety of the *H. rhamnoides* fruit extract, which showed no cytotoxicity on HT-29 monolayers, as expected for a traditionally used extract considered safe, and approved for use in dietary supplements without restrictions [16].

The main strength of this study is that to the best of our knowledge, one clinical trial on constipated children and few preclinical studies have hypothesized the use of *H. rhamnoides* fruit as a natural remedy for functional constipation. This study is the first randomized clinical trial to demonstrate the extract’s effectiveness in improving bowel function and reducing characteristic symptoms in adults suffering from chronic idiopathic constipation. Another strength of this study is its randomized, placebo-controlled design and the use of an EMA-recommended primary outcome. This robust design enables a rational comparison of the effects of *H. rhamnoides* fruit extract with those of the placebo group. Using SCBM as the primary outcome, together with a daily bowel function diary, helps minimize recall bias. However, the study has several limitations. It was monocentric, meaning it was conducted at a single center, which limits the generalizability of the findings to a broader population. Although 4 weeks is considered adequate under EFSA guidelines for assessing the efficacy of food supplements in constipation, this duration does not allow us to evaluate long-term effectiveness.

## 5. Conclusions

In conclusion, *H. rhamnoides* fruit extract, delivered through a food supplement in capsule form, proved effective in improving intestinal function in subjects with CIC. Compared to placebo, participants receiving *H. rhamnoides* fruit extract at the daily dose of 500 mg showed a statistically significant increase in weekly bowel movements, and a significant normalization of stool consistency. The improvement in intestinal function was associated with a reduction in intestinal discomfort, as subjects receiving the extract reported a statistically significant decrease in bloating, heaviness, and abdominal pain compared to the placebo group. This improvement in intestinal comfort was also associated with a non-significant trend toward enhanced mental component scores of the QoL. *H. rhamnoides* extract was well tolerated and did not cause any adverse effects in the study population. Based on these findings, *H. rhamnoides* extract appears to be an effective and well-tolerated option for managing functional constipation and may represent a promising alternative for further study alongside commonly used laxative medications and other food supplement plant-based ingredients.

## Figures and Tables

**Table 1:** Tentatively identified compounds by RP-UHPLC-ESI-Orbitrap-MS/MS analysis, including the retention time (min), molecular formula, experimental molecular ions, calculated mass error (Δm/z, ppm), MS/MS product ions.

Peak	RT (min)	Name	Formula	*m*/*z*	MS2	Ion	Error (ppm)	Class
***Positive***								
1	2.19	N-Acetylserotonin	C_12_H_14_N_2_O_2_	201.1021	160.0759; 184.0755	[M+H-H_2_O]^+^	−0.56	-
2	2.58	N-Acetylserotonin	C_12_H_14_N_2_O_2_	201.1021	160.0759; 184.0753	[M+H-H_2_O]^+^	−0.71	-
3	3.82	Quercetin 3*-O-*sophoroside-7-Orhamnoside	C_33_H_40_O_21_	773.2129	449.1072; 303.0498	[M+H]^+^	−0.77	Flavonoid
4	3.95	Isorhamnetin 3*-O-*sophoroside-7-*O*-rhamnoside	C_34_H_42_O_21_	787.228	463.1213; 317.0654	[M+H]^+^	−1.41	Flavonoid
5	4.13	Isorhamnetin 3*-O-*sophoroside-7-*O*-rhamnoside	C_34_H_42_O_21_	787.2278	463.1232; 317.0654	[M+H]^+^	−1.72	Flavonoid
6	4.36	Quercetin 3-glucoside-7-	C_27_H_30_O_16_	611.16	303.0498; 449.1069	[M+H]^+^	−0.63	Flavonoid
		rhamnoside						
7	4.77	Isorhamnetin 3*-O-*neohesperidoside	C_28_H_32_O_16_	625.1749	317.0654; 85.0283	[M+H]^+^	−1.38	Flavonoid
8	4.88	Quercetin 3-glucoside	C_21_H_20_O_12_	465.1021	303.0498	[M+H]^+^	−0.67	Flavonoid
9	5.21	Isorhamnetin 3*-O-*neohesperidoside	C_28_H_32_O_16_	625.1748	317.0654; 85.0283	[M+H]^+^	−1.45	Flavonoid
10	5.38	Isorhamnetin 3-glucoside	C_22_H_22_O_12_	479.1175	317.0655	[M+H]^+^	−0.94	Flavonoid
11	5.83	Kaempferol 3-glucoside	C_21_H_20_O_11_	449.1075	287.0549	[M+H]^+^	−0.32	Flavonoid
12	5.95	Isorhamnetin3*-O-β*-D-glucopyranoside-7*-O-*(2‴O-isovaleryl)-*β*-Dglucopyranoside	C_33_H_40_O_18_	725.2282	479.1184; 317.0654; 229.1542	[M+H]^+^	−0.36	Flavonoid
13	6.22	Isorhamnetin 3*-O-α*-L-rhamnopyranosyl-(1‴ → 6″)-*β*-D-glucopyranoside-7*-O-*(2”“O-isovaleryl)-*β*-D-glucopyranoside	C_39_H_50_O_22_	871.2855	317.0653; 229.1668; 625.1478	[M+H]^+^	−1.46	Flavonoid
14	6.39	Quercetin	C_15_H_10_O_7_	303.0496	285.0392; 229.1620; 165.0192	[M+H]^+^	−0.31	Flavonoid
15	6.62	Isorhamnetin 3-rhamnoside	C_22_H_22_O_11_	463.1227	317.0654	[M+H]^+^	−0.75	Flavonoid
16	7	Isorhamnetin 3*-O-β*-D-glucopyranoside-7*-O-*(3‴O-isovaleryl)-*α*-Lrhamnopyranoside	C_33_H_40_O_17_	709.2328	463.1221; 317.0653; 229.1771	[M+H]^+^	−1.22	Flavonoid
17	7.2	Isorhamnetin 3*-O-β*-D-glucopyranoside-7*-O-*(2‴O-isovaleryl)-*α*-Lrhamnopyranoside	C_33_H_40_O_18_	709.2328	46.1229; 317.0654; 229.1542	[M+H]^+^	1.35	Flavonoid
18	7.37	Isorhamnetin	C_16_H_12_O_7_	317.0652	302.0417; 285.0406; 229.1783; 274.0457; 257.0447	[M+H]^+^	−0.36	Flavonoid
19	7.9	Dehydrohytosphingosine	C_18_H_37_NO_3_	316.2843	60.0444; 280.2649	[M+H]^+^	−0.76	Sphingolipids
20	8.04	Dehydrohytosphingosine	C_18_H_37_NO_3_	316.2843	60.0443; 280.2633	[M+H]^+^	−1.1	Sphingolipids
21	8.27	PC (16:0)	C_25_H_52_NO_7_P	510.3186	184.0732; 104.1069; 309.2421; 492.3083	[M+H]^+^	−0.9	Glycerophosphocholines
22	0	Phytosphingosine	C_18_H_39_NO_3_	318.2999	60.0444; 282.2788; 300.2896	[M+H]^+^	−0.17	Sphingolipids
23	8.57	3-Oxoglycyrhetinicacid	C_30_H_44_O_4_	469.3305	451.3205; 423.3259; 405.3151	[M+H]^+^	−1.56	Triterpenoids
24	9.89	PC (16:0)	C_24_H_46_O_7_NP	492.3079	184.0732; 104.1069; 309.2427	[M+H-H_2_O]^+^	−0.3	Glycerophosphocholines
25	10.37	LysoPC (16:1)	C_24_H_48_O_7_NP	495.3233	184.0732; 104.1069; 311.2570	[M+H]^+^	−1.58	Glycerophosphocholines
26	10.43	LysoPE (16:1)	C_21_H_42_NO_7_P	452.2763	311.2579; 391.2235; 62.0600	[M+H]^+^	−1.76	Glycerophosphoethanolamines
27	10.58	LysoPC (16:1)	C_24_H_48_O_7_NP	494.3232	184.0732; 104.1069; 311.2596; 473.3126	[M+H]^+^	−1.89	Glycerophosphocholines
28	10.88	LysoPE (18:2)	C_23_H_44_NO_7_P	478.2921	337.2734; 306.2785; 62.0600	[M+H]^+^	−1.21	Glycerophosphoethanolamines
29	11.02	LysoPC (18:2)	C_26_H_50_O_7_NP	520.3386	184.0732; 104.1069; 258.1106; 502.3273	[M+H]^+^	−2.31	Glycerophosphocholines
30	11.49	LysoPC (16:0)	C_24_H_50_O_7_NP	496.339	184.0731; 104.1068; 313.2739; 478.3287; 258.1100	[M+H]^+^	−1.5	Glycerophosphocholines
31	11.82	LysoPC (18:1)	C_26_H_52_O_7_NP	522.3545	184.0731; 104.1069; 125.0000	[M+H]^+^	−1.77	Glycerophosphocholines
32	12.7	Oleanolaldehyde/Ursolaldehyde	C_30_H_48_O_2_	441.3722	423.3270; 405.3521; 395.3690; 187.1476	[M+H]^+^	−1.17	Triterpenoids
33	13.28	Coumaroylcorosolic acid/Coumaroylmaslinic acid (C_30_H_48_O_4_-CouA)	C_39_H_54_O_8_	619.3985	147.0439	[M+H]^+^	−1.22	Triterpenoids
34	13.35	Coumaroylcorosolic acid/Coumaroylmaslinic acid (C_30_H_48_O_4_-CouA)	C_39_H_54_O_9_	619.3985	147.0439	[M+H]^+^	−1.32	Triterpenoids
35	13.42	Coumaroylcorosolic acid/Coumaroylmaslinic acid (C_30_H_48_O_4_-CouA)	C_39_H_54_O_6_	619.3982	147.0439	[M+H]^+^	−1.91	Triterpenoids
36	13.52	Coumaroylcorosolic acid/Coumaroylmaslinic acid (C_30_H_48_O_4_-CouA)	C_39_H_54_O_7_	619.3984	147.044	[M+H]^+^	−1.22	Triterpenoids
37	13.55	Ursolic acid/Oleanolic acid	C_30_H_48_O_3_	457.367	439.3568; 411.3615; 393.3518; 203.1793; 187.1484	[M+H]^+^	−1.11	Triterpenoids
38	13.92	Oleanolic acid	C_30_H_47_O_2_	439.3564	393.3522; 203.1793; 187.1487	[M+H-H_2_O]^+^	−1.53	Triterpenoids
39	14.1	Oleanolic acid	C_30_H_47_O_3_	439.3567	393.3508; 203.1792; 187.1637	[M+H-H_2_O]^+^	−1.05	Triterpenoids
40	14.24	Coumaroylcorosolic acid/Coumaroylmaslinic acid (C_30_H_48_O_4_-CouA)	C_39_H_54_O_10_	619.3988	147.044	[M+H]^+^	−0.63	Triterpenoids
41	14.45	Betuline	C_30_H_50_O_2_	443.388	407.3673; 413.3786; 395.3684; 191.1794	[M+H]^+^	−0.76	Triterpenoids
42	14.64	Betuline	C_30_H_50_O_2_	425.3773	407.3694; 395.3684; 191.1795	[M+H-H_2_O]^+^	−0.54	Triterpenoids
43	15.32	cis-11-Eicosenamide	C_20_H_39_NO	310.3101	293.2837; 275.2732; 240.2318	[M+H]^+^	−0.84	Fatty amide
44	16	Betuline	C_30_H_50_O_2_	443.3878	425.3778; 407.3679; 413.3793; 395.3663; 191.1794	[M+H]^+^	−1.46	Triterpenoids
45	16.52	Erucamide	C_22_H_43_NO	338.3414	321.3152; 303.3046; 268.2647;	[M+H]^+^	−1.01	Fatty amide
***Negative***								
46	0.76	Quinic acid	C_7_H_12_O_6_	191.056	173.0455; 155.0357; 111.0452	[M-H]^−^	−0.75	Cyclohexanecarboxylic acid
47	0.86	Malic acid	C_4_H_6_O_5_	133.0143	115.0037; 71.0139; 89.0244	[M-H]^−^	−0.06	Dicarboxylic acid
48	3.28	Gastrodin A	C_19_H_28_O_12_	447.151	269.1033; 401.1458; 161.0456; 233.0664; 101.0245; 71.0139	[M-H]^−^	2.84	Glycosides
49	3.54	Everlastoside C	C_16_H_30_O_10_	427.1821	249.1346; 381.1767; 131.0350; 101.0245	[M+FA-H]^−^	2.49	Glycosides
50	4.73	Quercetin-3*-O-*rutinoside	C_27_H_30_O_16_	609.1465	300.0276; 301.0353; 151.0036; 178.9986	[M-H]^−^	2.12	Flavonoids
51	4.77	Isorhamnetin-3*-O-β*-D-glucopyranoside-7*-O-α*-L-rhamnopyranoside	C_28_H_32_O_16_	623.162	460.1012; 477.1039; 315.051; 357.0605	[M-H]^−^	2.62	Flavonoids
52	4.87	Quercetin-3*-O-*glucoside	C_21_H_20_O_12_	463.0882	300.0276; 151.0038	[M-H]^−^	2.76	Flavonoids
53	5.2	Isorhamnetin-3*-O-α*-L-rhamnopyranosyl-(1 → 6)-*β*-D-glucopyranoside	C_28_H_32_O_16_	623.1619	315.0510; 300.0275; 271.0266	[M-H]^−^	1.34	Flavonoids
54	5.38	Isorhamnetin-3*-O-β*-D-glucopyranoside	C_22_H_22_O_12_	477.1037	314.0434; 315.0508; 357.0607; 299.1674	[M-H]^−^	2.62	Flavonoids
55	6.38	Quercetin	C_15_H_10_O_7_	301.0354	151.0037; 178.9987; 121.029; 107.0137	[M-H]^−^	4.03	Flavonoids
56	6.64	Luteolin 7-*O*-(400-*O*-(E)-coumaroyl)-b-glucopyranoside	C_30_H_26_O_13_	593.1303	285.0403; 447.0931; 307.0823; 229.1822	[M-H]^−^	1.44	Flavonoids
57	7.37	Isorhamnetin	C_16_H_12_O_7_	315.0511	300.0278; 229.1713; 151.0036; 272.0320	[M-H]^−^	4.06	Flavonoids
58	10.09	DGMG (18:3)	C_34_H_56_O_14_	721.3658	277.2175; 397.1351; 235.0826; 415.1455	[M+FA-H]^−^	2.4	Glycerolipids
59	10.27	DGMG (16:1)	C_31_H_56_O_14_	697.3657	253.2174; 397.1349; 235.0823; 415.1455	[M+FA-H]^−^	2.31	Glycerolipids
60	10.42	LysoPE (16:1)	C_21_H_42_NO_7_ P	450.2625	253.2174; 196.0381; 140.0123; 78.9594	[M-H]^−^	1.94	Glycerophospholipids
61	10.56	LysoPC (16:1)	C_24_H_48_NO_7_ P	538.3151	253.2174; 224.0697; 478.2940; 78.9592; 373.4453	[M+FA-H]^−^	2.19	Glycerophospholipids
62	10.68	DGMG (18:2)	C_33_H_58_O_14_	723.3815	279.2332; 397.1348; 415.1453; 235.0821	[M+FA-H]^−^	2.36	Glycerolipids
63	10.86	LysoPE (18:2)	C_23_H_44_NO_7_P	476.2782	279.2331; 196.0381; 214.0493	[M+FA-H]^−^	2.23	Glycerophospholipids
64	10.95	MGMG (18:3)	C_27_H_46_O_9_	559.3126	277.2175; 253.0930; 235.0821; 101.0247	[M+FA-H]^−^	2.38	Glycerolipids
65	11.01	LysoPC (18:2)	C_26_H_50_NO_7_P	564.3312	279.2334; 224.0697	[M+FA-H]^−^	2.8	Glycerophospholipids
66	11.18	Palmitoleic-oleic	C_26_H_48_O_11_	535.3127	253.2175; 101.0246; 161.0458; 189.3665; 229.1627; 304.3327	[M-H]^−^	2.72	Di-fatty acid
67	11.48	LysoPC (16:0)	C_24_H_50_NO_7_P	540.3312	255.2331; 224.0697; 480.3098; 229.1720	[M+FA-H]^−^	2.7	Glycerophospholipids
68	11.63	Linoleic-oleic acid	C_28_H_50_O_11_	561.3286	279.2333; 253.0932; 235.0830	[M-H]^−^	2.87	Di-fatty acid
69	11.8	LysoPC (18:1)	C_26_H_52_NO_7_P	566.3468	281.2488; 224.0696; 506.3257; 78.9592; 402.9113	[M+FA-H]^−^	2.75	Glycerophospholipids
70	12.42	(2R)-3-(beta-D-Galactopyranosyloxy)-2-hydroxypropyl (9E)-9-octadecenoate	C_27_H_50_O_9_	563.3441	281.2486; 253.0929; 161.0459; 101.0246	[M+FA-H]^−^	2.6	-
71	13.72	Cordyglycoside A	C_25_H_44_O_8_	471.2964	71.0139; 281.2487; 115.0037; 453.2841	[M-H]^−^	0.02	Glycoside
72	15.11	LysoPI (18:3)	C_27_H_47_O_12_P	593.2738	277.2175; 152.9960; 241.0119; 315.0488; 413.2107	[M-H]^−^	2.7	Glycerophospholipids
73	16.13	LysoPI (16:1)	C_25_H_47_O_12_P	569.2737	253.2175; 152.9960; 241.0121; 315.0490; 389.2096	[M-H]^−^	2.39	Glycerophospholipids
74	19	Lauryl sulfate	C_12_H_26_O_4_S	265.1481	96.9602; 265.1480; 79.3549	[M-H]^−^	4.54	Alkyl sulfate
75	20.54	LysoPI (18:2)	C_27_H_49_O_12_P	595.2893	279.2332; 152.9960; 241.0120; 315.0490; 415.2251	[M-H]^−^	2.45	Glycerophospholipids

DGMG, digalactosylmonoacylglycerol; MGMG, monogalactosylmonoacylglycerol; PE, phosphoethanolamine; PC, phosphocholine; and PI, phosphoinositol.

**Table 2:** Baseline demographic and clinical data of the study population.

Characteristics of Enrolled Subjects	*H. rhamnoides* Group (*n* = 45)	Placebo Group (*n* = 45)
Mean age (years) ± SD	44.3 ± 14	43.8 ± 15
• Men	43.2 ± 16	44.0 ± 13
• Women	45.6 ± 13	43.6 ± 17
Gender		
• Men	20	25
• Women	25	20
Ethnicity	Caucasian	Caucasian

**Table 3:** Descriptive statistics (mean, standard deviation, and range) for the 11 response variables measured from time T0 to time T1 in the two experimental groups.

	*H. rhamnoides* Group		Placebo Group	
	T0	T1	T0	T1
SCBM	1.5 ± 0.5	2.6 ± 1	1.5 ± 0.5	1.5 ± 0.5
	(1–2)	(1–4)	(1–2)	(1–2)
BSFS	1.4 ± 0.5	3.5 ± 1.0	1.5 ± 0.5	1.7 ± 0.8
	(1–2)	(2–5)	(1–2)	(1–3)
Symptom A	3.1 ± 1.5	2.0 ± 0.8	3.0 ± 1.3	2.9 ± 1.5
	(1–5)	(1–3)	(1–5)	(1–5)
Symptom B	2.9 ± 1.5	2.0 ± 0.8	3.2 ± 1.5	2.9 ± 1.6
	(1–5)	(1–3)	(1–5)	(1–5)
Symptom C	2.8 ± 1.5	2.1 ± 0.8	2.8 ± 1.3	2.9 ± 1.5
	(1–5)	(1–3)	(1–5)	(1–5)
Symptom D	1.4 ± 0.5	2.0 ± 0.9	1.5 ± 0.5	2.2 ± 0.8
	(1–2)	(1–3)	(1–2)	(1–3)
Symptom E	3.0 ± 1.4	2.0 ± 0.9	2.6 ± 1.5	3.0 ± 1.3
	(1–5)	(1–3)	(1–5)	(1–5)
PCS	44.6 ± 6.3	42.6 ± 6.2	44.2 ± 6.3	44.4 ± 6.2
	(30.42–57.26)	(24.84–53.9)	(31.52–57.61)	(27.09–58.67)
MCS	35.2 ± 9.3	37.1 ± 7.5	37.3 ± 8.8	34.7 ± 7.4
	(16.05–58.09)	(11.21–51.57)	(17.69–55.4)	(16.28–51.67)
BMI	23.7 ± 3.3	23.7 ± 3.2	24.1 ± 3.2	24.1 ± 3
	(18–29)	(18–29)	(18–29)	(18–28)
WC	98 ± 5.2	98 ± 5.3	98.3 ± 5.5	97.5 ± 8.4
	(90–107)	(89–107)	(88–108)	(55–108)

SCBM: Number of bowel movements per week; BSFS: Bristol Stool Form Scale score; Symptom A: Bloating sensation; Symptom B: Abdominal distension; Symptom C: Heaviness; Symptom D: Flatulence; Symptom E: Abdominal pain; PCS: Quality-of-life score—physical component; MCS: Quality-of-life score—mental component; BMI: Body Mass Index; WC: Waist circumference.

**Table 4:** Results of the linear mixed-effects models (LMM) comparing treatment vs. placebo across the 11 response variables analyzed in this study.

Variable	F	d.o.f.	*p*
**SCBM**			
Measurement	31.345	1,174	**<0.001**
Treatment	29.172	1,174	**<0.001**
Sex	0.444	1,174	0.51
Age	3.28	1,174	0.072
Measurement × Treatment	28.888	1,174	**<0.001**
**BSFS**			
Measurement	112.504	1,174	**<0.001**
Treatment	63.446	1,174	**<0.001**
Sex	0.361	1,174	0.55
Age	6.14	1,174	**0.014**
Measurement × Treatment	69.577	1,174	**<0.001**
**Symptom A**			
Measurement	9.667	1,174	**<0.001**
Treatment	2.997	1,174	0.09
Sex	2.373	1,174	0.13
Age	1.989	1,174	0.16
Measurement × Treatment	6.471	1,174	**0.012**
**Symptom B**			
Measurement	8.46	1,174	**0.0041**
Treatment	7.755	1,174	**0.0059**
Sex	0.922	1,174	0.34
Age	0.842	1,174	0.36
Measurement × Treatment	2.894	1,174	0.091
**Symptom C**			
Measurement	2.291	1,174	0.13
Treatment	3.539	1,174	0.062
Sex	0.32	1,174	0.57
Age	1.57	1,174	0.21
Measurement × Treatment	5.421	1,174	**0.021**
**Symptom D**			
Measurement	48.861	1,88	**<0.001**
Treatment	3.235	1,86	0.075
Sex	0.647	1,86	0.42
Age	2.292	1,86	0.13
Measurement × Treatment	0.561	1,88	0.46
**Symptom E**			
Measurement	2.845	1,174	0.09
Treatment	2.798	1,174	0.1
Sex	0.265	1,174	0.61
Age	0.86	1,174	0.35
Measurement × Treatment	14.294	1,174	**<0.001**
**PCS**			
Measurement	1.03	1,174	0.31
Treatment	0.555	1,174	0.46
Sex	0.352	1,174	0.55
Age	0.241	1,174	0.62
Measurement × Treatment	1.428	1,174	0.23
**MCS**			
Measurement	0.092	1,174	0.76
Treatment	0.026	1,174	0.87
Sex	0.003	1,174	0.96
Age	1.69	1,174	0.2
Measurement × Treatment	3.463	1,174	0.064
**BMI**			
Measurement	0.043	1,88	0.84
Treatment	0.317	1,86	0.57
Sex	4.874	1,86	**0.03**
Age	0.196	1,86	0.66
Measurement × Treatment	0.043	1,88	0.84
**WC**			
Measurement	0.886	1,88	0.35
Treatment	0.006	1,86	0.94
Sex	0.7	1,86	0.41
Age	0.772	1,86	0.38
Measurement × Treatment	0.984	1,88	0.32

SCBM: Number of bowel movements per week; BSFS: Bristol Stool Form Scale score; Symptom A: Bloating sensation; Symptom B: Abdominal distension; Symptom C: Heaviness; Symptom D: Flatulence; Symptom E: Abdominal pain; PCS: Quality-of-life score—physical component; MCS: Quality-of-life score—mental component; BMI: Body Mass Index; WC: Waist circumference.

## Data Availability

The raw data supporting the conclusions of this article are stored at COMEGEN Soc. Coop. Sociale (Naples, Italy) and are available on request; further inquiries can be directed to the corresponding author.

## Abbreviations

The following abbreviations are used in this manuscript:

CIC	Chronic idiopathic constipation
UHPLC-HRMS/MS	Ultra-High-Performance Liquid Chromatography-High-Resolution Tandem Mass Spectrometry
SCBM	Spontaneous complete bowel movements
BSFS	Bristol Stool Form Scale
AQP-3	Aquaporin-3
SCFA	Short-chain fatty acids
EFSA	European Food Safety Authority
HIV	Human Immunodeficiency Virus
BMI	Body mass index
WC	Waist circumference
EMA	European Medicines Agency
LMM	Linear mixed-effects models
CRF	Case Report Form
QoL	Quality of life
SF-12	Short Form Health Survey-12
SUSAR	Suspected unexpected serious adverse reaction
ANOVA	Analysis of Variance
CONSORT PRO	CONsolidated Standards of Reporting Trials—Patient-Reported Outcomes
PCS	Physical Component Summary
MCS	Mental Component Summary
